# 4-Bromo-*N*-(diisopropoxyphosphor­yl)benzamide

**DOI:** 10.1107/S1600536809044523

**Published:** 2009-10-31

**Authors:** Christoph E. Strasser, Xia Sheng, Damir A. Safin, Helgard G. Raubenheimer, Robert C. Luckay

**Affiliations:** a Department of Chemistry and Polymer Science, University of Stellenbosch, Private Bag X1, Matieland, 7602, South Africa; b A. M. Butlerov Chemistry Institute, Kazan State University, Kremlevskaya Street 18, 420008 Kazan, Russian Federation

## Abstract

In the title compound, C_13_H_19_BrNO_4_P, the crystal structure is stabilized by inter­molecular N—H⋯O hydrogen bonds between the phosphoryl O atom and the amide N atom which link the mol­ecules into centrosymmetric dimers. These dimers are further packed into stacks along the *c* axis by inter­molecular C—H⋯O and C—H⋯π inter­actions.

## Related literature

For the synthesis, see: Safin, Sokolov, Baranov *et al.* (2008 ▶). For related structures, see: Chekhlov (1990 ▶); Safin *et al.* (2009 ▶); Safin, Sokolov, Nöth *et al.* (2008 ▶); Solov’ev *et al.* (1990 ▶). For the chemistry of phosphine derivatives of urea and thio­urea, see: Birdsall *et al.* (1999 ▶). For the use of bidentate organophospho­rus ligand systems, see: Crespo *et al.* (2004 ▶); Safin *et al.* (2006 ▶) and for the transport and extraction of metal ions, see: Luckay *et al.* (2009*a* ▶,*b* ▶).
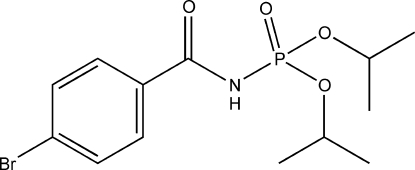


## Experimental

### 

#### Crystal data

C_13_H_19_BrNO_4_P
*M* *_r_* = 364.17
Monoclinic, 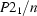

*a* = 8.611 (1) Å
*b* = 19.786 (3) Å
*c* = 9.849 (1) Å
β = 95.357 (2)°
*V* = 1670.7 (4) Å^3^
*Z* = 4
Mo *K*α radiation
μ = 2.57 mm^−1^
*T* = 100 K
0.32 × 0.07 × 0.05 mm

#### Data collection

Bruker APEX CCD area-detector diffractometer
Absorption correction: multi-scan (*SADABS*; Bruker, 2002 ▶) *T*_min_ = 0.494, *T*_max_ = 0.893
9035 measured reflections
3405 independent reflections
2604 reflections with *I* > 2σ(*I*)
*R*_int_ = 0.038

#### Refinement

*R*[*F* ^2^ > 2σ(*F* ^2^)] = 0.044
*wR*(*F* ^2^) = 0.120
*S* = 1.05
3405 reflections
185 parameters
H-atom parameters constrained
Δρ_max_ = 1.29 e Å^−3^
Δρ_min_ = −0.65 e Å^−3^

### 

Data collection: *SMART* (Bruker, 2002 ▶); cell refinement: *SAINT* (Bruker, 2003 ▶); data reduction: *SAINT*; program(s) used to solve structure: *SHELXS97* (Sheldrick, 2008 ▶); program(s) used to refine structure: *SHELXL97* (Sheldrick, 2008 ▶); molecular graphics: *X-SEED* (Barbour, 2001 ▶; Atwood & Barbour, 2003 ▶); software used to prepare material for publication: *X-SEED*.

## Supplementary Material

Crystal structure: contains datablocks I, global. DOI: 10.1107/S1600536809044523/lx2117sup1.cif

Structure factors: contains datablocks I. DOI: 10.1107/S1600536809044523/lx2117Isup2.hkl

Additional supplementary materials:  crystallographic information; 3D view; checkCIF report

## Figures and Tables

**Table 1:** Hydrogen-bond geometry (Å, °)

*D*—H⋯*A*	*D*—H	H⋯*A*	*D*⋯*A*	*D*—H⋯*A*
N1—H1⋯O1^i^	0.88	1.96	2.819 (3)	166
C3—H3⋯O4^ii^	0.95	2.29	3.213 (4)	163
C6—H6⋯O1^i^	0.95	2.48	3.241 (3)	137
C16—H16*C*⋯*Cg*^iii^	0.98	2.63	3.608 (4)	173

Symmetry codes: (i) 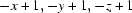; (ii) 
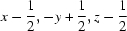
; (iii) 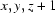. *Cg* is the centroid of the C1–C6 benzene ring.

**Fig. 1.**
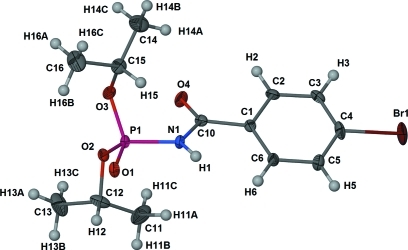
The molecular structure of the title compound with the atom numbering scheme. Displacement ellipsoids are drawn at the 50% probability level. H atoms are presented as small spheres of arbitrary radius.

**Fig. 2.**
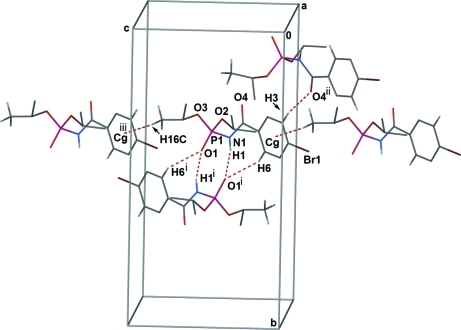
N—H···O, C—H···O and C—H···π interactions (dotted lines) in the crystal structure of the title compound. Cg denotes the ring centroids. Symmetry codes: (i) - x + 1, - y + 1, - z + 1; (ii) x - 1/2, - y + 1/2, z - 1/2; (iii) x, y, z + 1.

## supplementary crystallographic information

### Comment

The chemistry of phosphine derivatives of urea and thiourea was first studied during the 1960 s (Birdsall *et al.*, 1999). Subsequently, related bidentate organophosphorus ligand systems were developed to form *R*^1^C(*X*)NHP*R*_2_ and their derivatives (Safin *et al.*, 2006). Different *R*^1^C(X)NHP(Y)*R*^2^*R*^3^ (*R*^1^ = RNH or NZ_2_ with Z = H, alkyl or aryl; *R*^2^, *R*^3^ = alkyl, aryl, alkoxy or aryloxy; X, Y = O, S, Se) have been reported (Crespo *et al.*, 2004). These types of ligands have recently been used successfully as ionophores for the transport and extraction of a number of metal ions (Luckay *et al.*, 2009*a*, 2009*b*). Here we report the crystal structure of the title compound (I) (Fig. 1).

The crystal structure is stabilized by intermolecular N—H···O hydrogen bonds between the phosphoryl O atom and the amide N atom which link the molecules into centrosymmetric dimers (Table 1 and Fig. 2). These dimers are further packed into stacks along the *c* axis by intermolecular C—H···O and C—H···π interactions; the first between the benzene H atom and the oxygen of the C═O unit, with a C3—H3···O4^ii^, the second between the benzene H atom and the oxygen of the P═O unit, with a C6—H6···O1^i^, the third between the methyl H atom of the isopropyl group and the benzene ring, with a C16—H16C···Cg^iii^ (Cg is the centroid of the C1–C6 benzene ring), respectively (Table 1 and Fig. 2).

### Experimental

4-bromo-N-(diisopropoxyphosphoryl)benzthioamide was prepared according to the procedure of Safin *et al.* (2009). This ligand and one equivalent of copper(I) iodide was dissolved in acetone and heated to 50 °C for 2 hours. The colourless powder obtained was dissolved in a minimal quantity of THF and allowed to slowly evaporate. After 6 days, colourless needles were deposited. The hydrolysis of the thione group group was most likely caused by the presence of moisture in the solvents as well as the presence of the Cu^+^ ion.

### Refinement

All H atoms were positioned geometrically (C—H = 0.95, 1.00 and 0.98 Å for aromatic CH, alkyl CH and CH_3_ groups, respectively; N—H = 0.88 Å) and constrained to ride on their parent atoms. *U*_iso_(H) values were set at 1.2 times *U*_eq_(C,*N*) except for methyl groups where *U*_iso_(H) was set at 1.5 times *U*_eq_(C).

The largest residual electron density peak of 1.29 e Å^-3^ is located 0.93 Å next to Br1.

### Crystal data

**Table:**

C_13_H_19_BrNO_4_P	*F*(000) = 744
*M**_r_* = 364.17	*D*_x_ = 1.448 Mg m^−^^3^
Monoclinic, *P*2_1_/*n*	Mo *K*α radiation, λ = 0.71073 Å
Hall symbol: -P 2yn	Cell parameters from 2372 reflections
*a* = 8.611 (1) Å	θ = 2.3–26.3°
*b* = 19.786 (3) Å	µ = 2.57 mm^−^^1^
*c* = 9.849 (1) Å	*T* = 100 K
β = 95.357 (2)°	Needle, colourless
*V* = 1670.7 (4) Å^3^	0.32 × 0.07 × 0.05 mm
*Z* = 4	

### Data collection

**Table:**

Bruker APEX CCD area-detector diffractometer	3405 independent reflections
Radiation source: fine-focus sealed tube	2604 reflections with *I* > 2σ(*I*)
graphite	*R*_int_ = 0.038
ω scans	θ_max_ = 26.5°, θ_min_ = 2.1°
Absorption correction: multi-scan (*SADABS*; Bruker, 2002)	*h* = −10→9
*T*_min_ = 0.494, *T*_max_ = 0.893	*k* = −24→24
9035 measured reflections	*l* = −8→12

### Refinement

**Table:**

Refinement on *F*^2^	Primary atom site location: structure-invariant direct methods
Least-squares matrix: full	Secondary atom site location: difference Fourier map
*R*[*F*^2^ > 2σ(*F*^2^)] = 0.044	Hydrogen site location: difference Fourier map
*wR*(*F*^2^) = 0.120	H-atom parameters constrained
*S* = 1.05	*w* = 1/[σ^2^(*F*_o_^2^) + (0.0687*P*)^2^] where *P* = (*F*_o_^2^ + 2*F*_c_^2^)/3
3405 reflections	(Δ/σ)_max_ = 0.001
185 parameters	Δρ_max_ = 1.29 e Å^−^^3^
0 restraints	Δρ_min_ = −0.65 e Å^−^^3^

### Special details

**Table:**

Geometry. All esds (except the esd in the dihedral angle between two l.s. planes) are estimated using the full covariance matrix. The cell esds are taken into account individually in the estimation of esds in distances, angles and torsion angles; correlations between esds in cell parameters are only used when they are defined by crystal symmetry. An approximate (isotropic) treatment of cell esds is used for estimating esds involving l.s. planes.
Refinement. Refinement of F^2^ against ALL reflections. The weighted R-factor wR and goodness of fit S are based on F^2^, conventional R-factors R are based on F, with F set to zero for negative F^2^. The threshold expression of F^2^ > 2sigma(F^2^) is used only for calculating R-factors(gt) etc. and is not relevant to the choice of reflections for refinement. R-factors based on F^2^ are statistically about twice as large as those based on F, and R- factors based on ALL data will be even larger.

### Fractional atomic coordinates and isotropic or equivalent isotropic displacement parameters (Å^2^)

**Table:**

	*x*	*y*	*z*	*U*_iso_*/*U*_eq_	
Br1	−0.02989 (4)	0.39671 (2)	−0.17708 (4)	0.03600 (16)	
P1	0.63994 (9)	0.40142 (4)	0.52752 (8)	0.01485 (19)	
O1	0.6535 (2)	0.46798 (10)	0.5943 (2)	0.0195 (5)	
O2	0.7917 (2)	0.37315 (10)	0.4748 (2)	0.0174 (5)	
O3	0.5980 (2)	0.34095 (10)	0.6189 (2)	0.0190 (5)	
O4	0.5279 (3)	0.29938 (10)	0.3200 (2)	0.0260 (5)	
N1	0.5031 (3)	0.40726 (12)	0.3964 (2)	0.0161 (5)	
H1	0.4524	0.4457	0.3836	0.019*	
C1	0.3453 (3)	0.36868 (15)	0.1896 (3)	0.0160 (6)	
C2	0.2722 (4)	0.31254 (15)	0.1264 (3)	0.0201 (7)	
H2	0.2994	0.2684	0.1583	0.024*	
C3	0.1603 (3)	0.32078 (16)	0.0175 (3)	0.0217 (7)	
H3	0.1095	0.2827	−0.0254	0.026*	
C4	0.1240 (4)	0.38516 (17)	−0.0273 (3)	0.0233 (7)	
C5	0.1961 (4)	0.44202 (16)	0.0329 (3)	0.0228 (7)	
H5	0.1702	0.4860	−0.0007	0.027*	
C6	0.3062 (3)	0.43314 (15)	0.1425 (3)	0.0177 (6)	
H6	0.3556	0.4714	0.1860	0.021*	
C10	0.4653 (3)	0.35442 (15)	0.3062 (3)	0.0166 (6)	
C11	0.8492 (5)	0.4199 (2)	0.2571 (4)	0.0480 (11)	
H11C	0.8381	0.3738	0.2210	0.072*	
H11B	0.9284	0.4440	0.2107	0.072*	
H11A	0.7491	0.4435	0.2414	0.072*	
C12	0.8981 (4)	0.41721 (17)	0.4076 (4)	0.0287 (8)	
H12	0.8952	0.4638	0.4469	0.034*	
C13	1.0587 (4)	0.3875 (2)	0.4397 (4)	0.0439 (11)	
H13A	1.0828	0.3848	0.5388	0.066*	
H13B	1.1359	0.4162	0.4007	0.066*	
H13C	1.0618	0.3420	0.4005	0.066*	
C14	0.3657 (4)	0.28522 (19)	0.6749 (4)	0.0366 (9)	
H14A	0.3324	0.2823	0.5772	0.055*	
H14B	0.2737	0.2884	0.7262	0.055*	
H14C	0.4256	0.2447	0.7036	0.055*	
C15	0.4659 (4)	0.34681 (17)	0.7022 (3)	0.0246 (7)	
H15	0.4038	0.3879	0.6732	0.030*	
C16	0.5304 (5)	0.3551 (2)	0.8481 (3)	0.0445 (11)	
H16A	0.5854	0.3137	0.8792	0.067*	
H16C	0.4449	0.3637	0.9048	0.067*	
H16B	0.6033	0.3932	0.8556	0.067*	

### Atomic displacement parameters (Å^2^)

**Table:**

	*U*^11^	*U*^22^	*U*^33^	*U*^12^	*U*^13^	*U*^23^
Br1	0.0283 (2)	0.0495 (3)	0.0267 (2)	0.00590 (16)	−0.01544 (16)	−0.00825 (16)
P1	0.0128 (4)	0.0175 (4)	0.0137 (4)	0.0027 (3)	−0.0017 (3)	−0.0004 (3)
O1	0.0173 (11)	0.0195 (11)	0.0204 (11)	0.0039 (9)	−0.0046 (9)	−0.0037 (9)
O2	0.0118 (10)	0.0202 (11)	0.0200 (11)	0.0009 (8)	0.0004 (8)	0.0004 (9)
O3	0.0174 (11)	0.0204 (11)	0.0197 (11)	0.0047 (9)	0.0043 (9)	0.0039 (9)
O4	0.0309 (13)	0.0168 (12)	0.0285 (13)	0.0061 (10)	−0.0071 (10)	0.0023 (9)
N1	0.0173 (13)	0.0148 (13)	0.0153 (13)	0.0027 (10)	−0.0040 (10)	−0.0011 (9)
C1	0.0156 (15)	0.0201 (16)	0.0125 (14)	−0.0008 (12)	0.0025 (12)	−0.0010 (12)
C2	0.0213 (16)	0.0184 (16)	0.0209 (16)	−0.0074 (12)	0.0024 (13)	0.0005 (12)
C3	0.0171 (16)	0.0243 (17)	0.0238 (17)	−0.0073 (13)	0.0028 (13)	−0.0050 (13)
C4	0.0150 (16)	0.040 (2)	0.0140 (16)	0.0005 (13)	−0.0025 (12)	−0.0076 (13)
C5	0.0240 (17)	0.0236 (17)	0.0196 (16)	0.0043 (13)	−0.0035 (13)	−0.0005 (13)
C6	0.0188 (16)	0.0163 (15)	0.0172 (15)	−0.0003 (12)	−0.0023 (12)	−0.0023 (12)
C10	0.0192 (15)	0.0174 (16)	0.0133 (15)	−0.0025 (12)	0.0026 (12)	−0.0016 (11)
C11	0.039 (2)	0.062 (3)	0.045 (3)	0.012 (2)	0.0156 (19)	0.027 (2)
C12	0.0242 (18)	0.0203 (17)	0.044 (2)	−0.0065 (14)	0.0148 (16)	−0.0082 (15)
C13	0.0169 (19)	0.077 (3)	0.039 (2)	−0.0014 (18)	0.0074 (17)	−0.010 (2)
C14	0.0266 (19)	0.040 (2)	0.045 (2)	−0.0015 (16)	0.0130 (16)	0.0000 (17)
C15	0.0194 (17)	0.0299 (18)	0.0257 (17)	0.0053 (14)	0.0082 (13)	0.0019 (14)
C16	0.036 (2)	0.077 (3)	0.0219 (19)	−0.011 (2)	0.0110 (16)	−0.0021 (19)

### Geometric parameters (Å, °)

**Table:**

Br1—C4	1.902 (3)	C6—H6	0.9500
P1—O1	1.472 (2)	C11—C12	1.504 (5)
P1—O2	1.555 (2)	C11—H11C	0.9800
P1—O3	1.560 (2)	C11—H11B	0.9800
P1—N1	1.669 (2)	C11—H11A	0.9800
O2—C12	1.466 (4)	C12—C13	1.509 (5)
O3—C15	1.468 (4)	C12—H12	1.0000
O4—C10	1.217 (4)	C13—H13A	0.9800
N1—C10	1.390 (4)	C13—H13B	0.9800
N1—H1	0.8800	C13—H13C	0.9800
C1—C6	1.388 (4)	C14—C15	1.503 (5)
C1—C2	1.394 (4)	C14—H14A	0.9800
C1—C10	1.498 (4)	C14—H14B	0.9800
C2—C3	1.383 (4)	C14—H14C	0.9800
C2—H2	0.9500	C15—C16	1.500 (4)
C3—C4	1.374 (4)	C15—H15	1.0000
C3—H3	0.9500	C16—H16A	0.9800
C4—C5	1.391 (4)	C16—H16C	0.9800
C5—C6	1.380 (4)	C16—H16B	0.9800
C5—H5	0.9500		
			
O1—P1—O2	115.92 (12)	H11C—C11—H11B	109.5
O1—P1—O3	116.21 (12)	C12—C11—H11A	109.5
O2—P1—O3	99.42 (11)	H11C—C11—H11A	109.5
O1—P1—N1	107.74 (12)	H11B—C11—H11A	109.5
O2—P1—N1	108.74 (12)	O2—C12—C11	109.7 (3)
O3—P1—N1	108.36 (12)	O2—C12—C13	105.8 (3)
C12—O2—P1	121.12 (19)	C11—C12—C13	112.8 (3)
C15—O3—P1	119.68 (18)	O2—C12—H12	109.5
C10—N1—P1	123.3 (2)	C11—C12—H12	109.5
C10—N1—H1	118.4	C13—C12—H12	109.5
P1—N1—H1	118.4	C12—C13—H13A	109.5
C6—C1—C2	119.8 (3)	C12—C13—H13B	109.5
C6—C1—C10	123.9 (3)	H13A—C13—H13B	109.5
C2—C1—C10	116.3 (3)	C12—C13—H13C	109.5
C3—C2—C1	120.3 (3)	H13A—C13—H13C	109.5
C3—C2—H2	119.8	H13B—C13—H13C	109.5
C1—C2—H2	119.8	C15—C14—H14A	109.5
C4—C3—C2	118.6 (3)	C15—C14—H14B	109.5
C4—C3—H3	120.7	H14A—C14—H14B	109.5
C2—C3—H3	120.7	C15—C14—H14C	109.5
C3—C4—C5	122.3 (3)	H14A—C14—H14C	109.5
C3—C4—Br1	118.8 (2)	H14B—C14—H14C	109.5
C5—C4—Br1	119.0 (3)	O3—C15—C16	107.9 (3)
C6—C5—C4	118.5 (3)	O3—C15—C14	107.3 (3)
C6—C5—H5	120.7	C16—C15—C14	114.5 (3)
C4—C5—H5	120.7	O3—C15—H15	109.0
C5—C6—C1	120.4 (3)	C16—C15—H15	109.0
C5—C6—H6	119.8	C14—C15—H15	109.0
C1—C6—H6	119.8	C15—C16—H16A	109.5
O4—C10—N1	121.8 (3)	C15—C16—H16C	109.5
O4—C10—C1	121.4 (3)	H16A—C16—H16C	109.5
N1—C10—C1	116.8 (3)	C15—C16—H16B	109.5
C12—C11—H11C	109.5	H16A—C16—H16B	109.5
C12—C11—H11B	109.5	H16C—C16—H16B	109.5
			
O1—P1—O2—C12	−40.8 (2)	Br1—C4—C5—C6	179.2 (2)
O3—P1—O2—C12	−166.1 (2)	C4—C5—C6—C1	1.0 (5)
N1—P1—O2—C12	80.7 (2)	C2—C1—C6—C5	−0.4 (4)
O1—P1—O3—C15	49.5 (2)	C10—C1—C6—C5	178.9 (3)
O2—P1—O3—C15	174.7 (2)	P1—N1—C10—O4	2.0 (4)
N1—P1—O3—C15	−71.9 (2)	P1—N1—C10—C1	−177.1 (2)
O1—P1—N1—C10	176.7 (2)	C6—C1—C10—O4	−158.7 (3)
O2—P1—N1—C10	50.3 (3)	C2—C1—C10—O4	20.6 (4)
O3—P1—N1—C10	−56.8 (3)	C6—C1—C10—N1	20.4 (4)
C6—C1—C2—C3	−0.4 (4)	C2—C1—C10—N1	−160.3 (3)
C10—C1—C2—C3	−179.7 (3)	P1—O2—C12—C11	−88.3 (3)
C1—C2—C3—C4	0.5 (5)	P1—O2—C12—C13	149.8 (2)
C2—C3—C4—C5	0.1 (5)	P1—O3—C15—C16	−106.3 (3)
C2—C3—C4—Br1	−180.0 (2)	P1—O3—C15—C14	129.8 (2)
C3—C4—C5—C6	−0.9 (5)		

### Hydrogen-bond geometry (Å, °)

**Table:**

*D*—H···*A*	*D*—H	H···*A*	*D*···*A*	*D*—H···*A*
N1—H1···O1^i^	0.88	1.96	2.819 (3)	166
C3—H3···O4^ii^	0.95	2.29	3.213 (4)	163
C6—H6···O1^i^	0.95	2.48	3.241 (3)	137
C16—H16C···Cg^iii^	0.98	2.63	3.608 (4)	173

Symmetry codes: (i) −*x*+1, −*y*+1, −*z*+1; (ii) *x*−1/2, −*y*+1/2, *z*−1/2; (iii) *x*, *y*, *z*+1.
